# A randomized single blind crossover trial comparing leather and commercial wrist splints for treating chronic wrist pain in adults

**DOI:** 10.1186/1471-2474-10-129

**Published:** 2009-10-21

**Authors:** Jill Thiele, Rachel Nimmo, Wendy Rowell, Stephen Quinn, Graeme Jones

**Affiliations:** 1 Occupational Therapy Department, Royal Hobart Hospital, Hobart, Australia; 2 Menzies Research Institute, University of Tasmania, Hobart, Australia

## Abstract

**Background:**

To compare the effectiveness of a custom-made leather wrist splint (LS) with a commercially available fabric splint (FS) in adults with chronic wrist pain.

**Methods:**

Participants (N = 25, mean age = 54) were randomly assigned to treatment order in a 2-phase crossover trial. Splints were worn for 2 weeks, separated by a one-week washout period. Outcomes were assessed at baseline and after each splint phase using the Australian/Canadian Osteoarthritis Hand Index (AUSCAN), the Canadian Occupational Performance Measure (COPM) and Jamar dynamometer by an observer blinded to treatment allocation.

**Results:**

Both styles of wrist splint significantly reduced pain (effect size LS 0.79, FS 0.43), improved hand function and increased grip strength compared to baseline (all p < 0.05) with no increase in wrist stiffness. There was a consistent trend for the LS to be superior to the FS but this was statistically significant only for patient perceived occupational performance (p = 0.008) and satisfaction (p = 0.015). Lastly, 72% of patients preferred the custom-made leather splint compared to the commercially available splint.

**Conclusion:**

Leather wrist splints were superior to a commercially available fabric splint for the short-term relief of pain and dysfunction.

## Background

Chronic wrist pain is a common clinical presentation, usually as a result of osteoarthritis (OA) or the inflammatory process of rheumatoid arthritis (RA). Patients with chronic wrist pain benefit from a multi-disciplinary approach incorporating pharmacotherapy and surgical opinion along with conservative Physical and Occupational therapies. Occupational therapists provide patient education regarding joint protection techniques, provide assistive devices and hand splints with the goal of preserving and optimizing hand function [1].

Traditionally wrist splints have been prescribed on the presumption that stabilizing the wrist joint allows inflamed joints to rest, reduces swelling, alleviates pain and improves hand function [2]. The efficacy of wrist splints have been reviewed with studies comparing many different styles, including commercially available fabric styles that are elasticized with a palmar metal insert, and custom-made thermoplastic splints.

Research on the effects of wrist splinting remains inconclusive with regards to the benefits and potential adverse effects of splinting. A Cochrane review examining Occupational therapy in management of RA, reported that limited evidence exists for the use of wrist splints to reduce pain in patients with RA[3]. This review cites Nordenskiold's [4] and Pagnotta's [5] studies that found a reduction in pain with the use of working wrist splints. An earlier Cochrane review of the use of splints in the management of RA found insufficient evidence to support a reduction in pain with the use of wrist splints [2]. Most recently, a crossover trial compared two types of commercially available fabric splints and a custom-made leather splint, concluding that all splints significantly reduced pain after four weeks of use with the leather splint being the most effective at diminishing pain [6].

The recent Cochrane review demonstrated a trend to increases in grip strength when patients with RA are splinted [3]. Pinch and power grip strength significantly improved with splinting in the study by Haskett [6], and a 25% improvement in grip strength was reported by Kjeken [7].

Research has not conclusively demonstrated a functional benefit of wrist splinting in OA or RA. Data regarding the impact of splint wearing on hand dexterity is inconclusive with two review articles reporting trends that dexterity is compromised [2,3], whilst Haskett's study found no negative impact on dexterity [6]. Importantly, studies by Pagnotta [8] and Haskett [6] indicate trends of improvement in patient perceived function during splint wear. O'Brien's study [9] suggests that hand strengthening exercises in patients with RA is related to improvements in hand function. However, causal relationships of improved hand function and improvement in grip strength or reduction in pain have yet to be conclusively demonstrated in the literature.

In our location we observed a clinical benefit of custom-made leather wrist splints for patients with chronic wrist pain, with many patients anecdotally reporting an increase in hand function and decrease in pain. Absence of the palmar metal insert and the "skin like" movement of the leather splint were postulated to allow limited wrist movement in combination with mechanical stability thus permitting improved functional performance. The objective of this study, therefore, was to compare the effectiveness of a custom-made leather wrist splint with a commercially available fabric splint on pain and hand function in adults with chronic wrist pain.

## Methods

**Figure 1:**
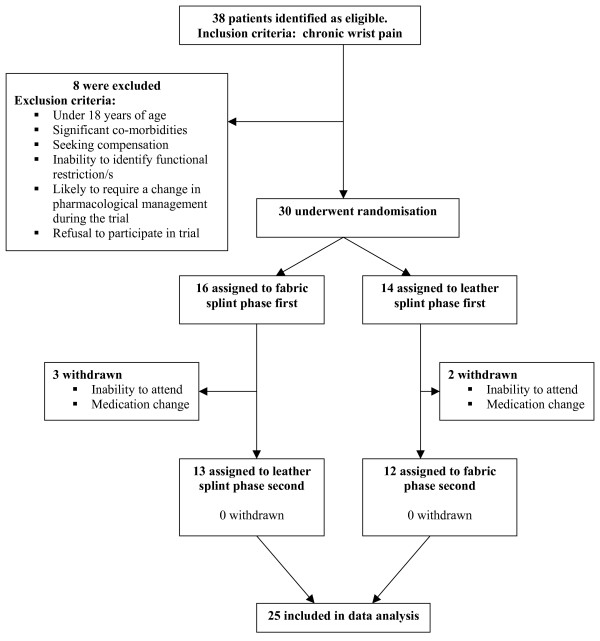
**Enrolment and follow-up of study participants**.

The setting was a tertiary referral public hospital clinic in Southern Tasmania, Australia. Patients were referred to the outpatient occupational therapy department, by rheumatologists. The inclusion criterion was a diagnosis of chronic wrist pain impairing functional activity. Potential participants were excluded if they were under 18 years of age, had significant co-morbidities (i.e. carpal tunnel, primary 1^st ^carpometocarpal joint pain), were seeking compensation, were unable to identify functional restrictions or were likely to require a change in pharmacological management during the trial. Diagrammatic representation of patient enrollment and follow-up is presented in Figure 1. The Royal Hobart Hospital Research Foundation Ethics Committee approved the study and written informed consent was obtained from all participants.

**Figure 2:**
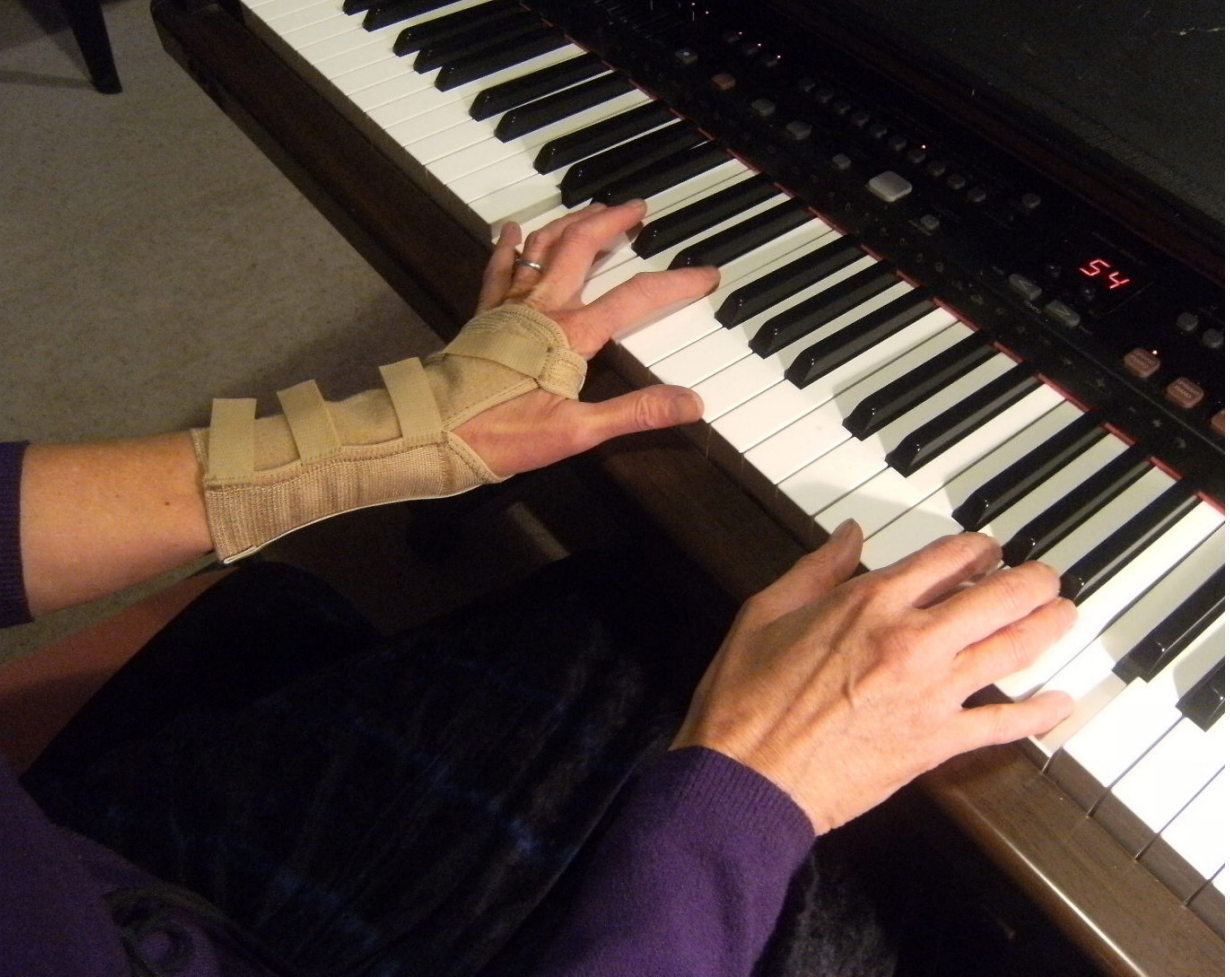
**Futuro^© ^wrist splint**.

**Figure 3:**
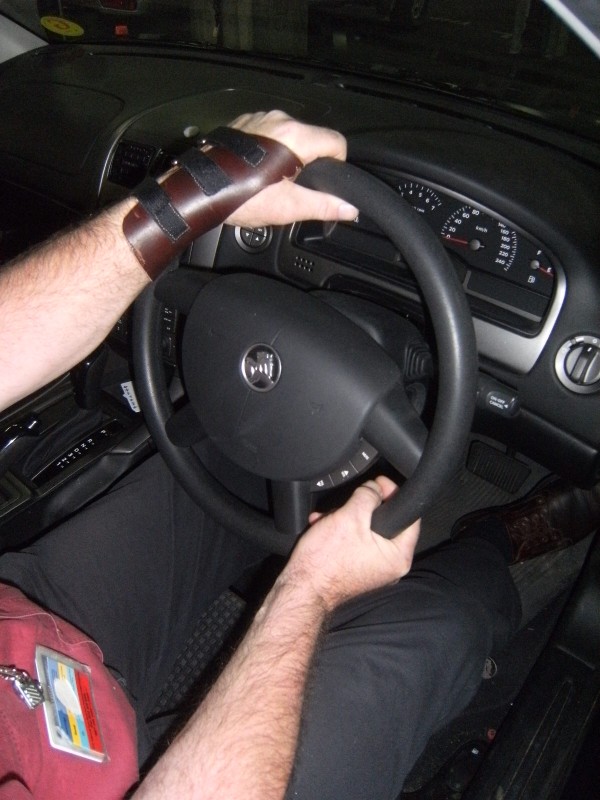
**Custom-made leather wrist splint**.

A crossover design was used, with consecutively referred patients randomly allocated to the two different treatment sequences using opaque sealed envelopes. The treatment consisted of a 2-week period of splint wear, followed directly by a one-week washout period. Plaster positive casts were made of the patients' painful wrist (wrist positioned in 15-20 degrees of extension) by the one occupational therapist. Patients who could not achieve this wrist position due to pain were cast in less than 15 degrees wrist extension. Where both wrists were affected the one with the greatest pain was splinted. Two different splint designs were used in this study. All splints were fitted by one occupational therapist. The Futuro^© ^splint is commercially available and is a circumferential elasticized fabric wrist brace, reinforced with a palmar metal bar that restricts wrist range of movement and provides stability (Figure 2). This fabric splint (FS) is fastened with four Velcro straps on the dorsum, comes in three sizes and was fitted to the participants according to manufacturers instructions. No modifications were made to the fabric splint. The custom leather splint (LS) was molded from a single layer of 3 mm embossing leather onto the plaster positive. It was made in a gauntlet style and fastened with four Velcro straps through D-rings on the dorsum (Figure 3). No metal bar was incorporated into the palmar surface. Patients were advised on the appropriate care and use of the splint, and were instructed to use the splint during periods of pain and discomfort. At the end of the trial, participants were given both splints free of charge. The total duration of the trial was 5 weeks.

Baseline evaluation included the collection of demographic data (age, gender, diagnosis, duration of disease, hand dominance) summarized in Table 1. The outcome measures to assess for wrist pain, stiffness, hand function, grip strength and patient perceived performance and satisfaction are described below. These outcomes were assessed at baseline and at the end of each two-week splint phase. An independent assessor (WR) completed all outcome measures and was blinded to the splint style and sequence. Patients using wrist splints prior to the beginning of the study undertook a two-week washout period prior to their first baseline measurement.

**Table 1:** Characteristics of the participants including age, gender, diagnosis, disease duration, and details of wrist splinted.

**Characteristics** **N=25**	**Data** **Mean (range or %)**
**Age **(years)	54 (18-82)

Male	12 (48%)

Female	13 (52%)

**Dominant wrist splinted**	22 (88%)

**Non-dominant wrist splinted**	3 (12%)

**Diagnosis:**	
OA	6 (24%)
RA	17 (68%)
Other inflammatory	2 (8%)

**Duration of disease **(years)	15 (1-58)

Data provided as mean with range or percentage of total number of participants.

Power grip strength (kg of force) was measured using a calibrated Jamar dynamometer. Grip strength was assessed using standardized technique and instruction, using the second grip position (patient seated, arms unsupported, elbow flexed at 90 degrees, neutral forearm rotation and wrist in 0-30 degrees of extension). All measurements were taken without splints applied. The Jamar dynamometer is considered to be a reliable and valid instrument for measuring handgrip strength when calibrated and using standard positioning and instructions [10].

General hand function, stiffness and pain were measured using the Australian/Canadian Osteoarthritis Hand Index VA 3.0 (AUSCAN). This tool uses a 10 cm visual analogue scale (VAS) scale where patients report on pain, stiffness and functional difficulty in set activities during the previous 48 hours. The VAS is anchored with 1 = no pain and 10 = extreme pain. The AUSCAN is reliable and valid for use in clinic patients with OA, and has also been demonstrated to have similar levels of reliability and validity when used in a community dwelling population of adults with hand problems [11].

The Canadian Occupational Performance Measure (COPM) provides a measure of patient self-perceived occupational performance in activities of daily living (ADL) [12]. It is a standardized assessment with specific methods for administering and scoring, however it is not norm-referenced, as it aims to describe individual subjective experiences [13]. It is reported to be a valid and reliable assessment tool [14]. The COPM has demonstrated concurrent criterion validity and sensitivity to change when used as an outcome measure in a pain management program [15]. Patient selected tasks are evaluated using a 10 cm VAS scale anchored with 1 = very unhappy with performance/not satisfied at all and 10 = extremely happy with performance/satisfied.

Prior to the assessment of outcome measures, patients were asked if they had undergone any changes to their pharmacotherapy regimen, to identify potential exclusions. At the conclusion of the trial patients were asked by an independent assessor to identify their preference for the leather splint, fabric splint or neither splint.

Differences in the effect of the LS and FS were compared to baseline and also between the two splint styles on all outcome measures. These were calculated using paired *t*-tests for follow-up baseline comparisons and unpaired *t*-tests for treatment comparison, with statistical significance set at *p *= 0.05 (two tailed). All analyses were performed on SPSS version 16 (Chicago Illinois).

## Results

Of the 38 patients identified, 4 were deemed ineligible and 4 declined to participate. 5 patients were withdrawn from the study following the first phase due to inability to attend or change in medication regimen. The characteristics of the sample (N = 25) are shown in Table 1. Table 2 presents the means of all outcome variables at baseline and following each splint phase. Table 3 presents the results of the comparison between the fabric and leather splints on all outcome measures.

**Table 2:** AUSCAN, COPM and grip strength measures at baseline, and after each splint phase, with P values.

	**Outcome measure**	**Baseline**	**Fabric splint (FS)**	**P**	**Leather splint (LS)**	**P**
**AUSCAN**	**Pain**	26 (11.5)	21 (11.55)	0.014	16.9 (9.44)	0.001
	
	**Stiffness**	4.6 (2.7)	3.3 (2.4)	0.53	3 (2.3)	0.02
	
	**Function**	54.4 (19.5)	43.1 (18.55)	0.014	39.4 (16.5)	<0.001

**COPM**	**Performance**	3.9 (1.0)	5.6 (1.9)	<0.001	6.4 (1.4)	<0.001
	
	**Satisfaction**	3.8 (1.2)	5.8 (1.9)	<0.001	6.4 (1.5)	<0.001

	**Grip strength kg**	17.1 (11.9)	20.6 (12.9)	<0.001	21.8 (12.8)	<0.001

Data reported as mean (standard deviation). P values reflect significance of the difference between splint and baseline using paired *t*-tests (2-tailed). AUSCAN scored on 10 cm VAS. COPM reported as average score of tasks selected using a 10 cm VAS.

**Table 3:** Pair-wise comparison of fabric splint and leather splint on AUSCAN and COPM and grip strength measures.

	**Outcome measure**	**Mean difference**	**SD**	**P**
**AUSCAN**	**Pain**	4.1	13.9	.149
	
	**Stiffness**	.29	3.7	.694
	
	**Function**	3.7	16.9	.287

**COPM**	**Performance**	-0.8	1.4	.008
	
	**Satisfaction**	-.68	1.3	.015

	**Grip strength, kg**	-1.3	3.87	.107

P values reflect significance of the difference between splints using paired *t*-tests (2-tailed). AUSCAN scored on 10 cm VAS. COPM reported as average score of tasks selected using a 10 cm VAS.

Both splints achieved a statistically significant decrease in pain with a trend to superiority for the LS. Again, both splints achieved a statistically significant improvement in function (AUSCAN), however there was no significant difference between the splints on this measure. The baseline score for stiffness was a mean 4.6 cm on VAS. There was a significant reduction in stiffness with the LS, but not with the FS. There was no significant difference in effect between the leather and fabric splints with regards to stiffness. The baseline measurement for grip strength was a mean of 17.1 kg. Both splints achieved a statistically significant increase in grip strength, with no significant difference between the splints.

COPM results were reported as mean scores due to a lack of precision when individual scores were used [16]. Baseline measurement for perceived performance was a mean 3.9 cm on VAS and perceived satisfaction was a mean 3.8 cm on VAS. Both achieved statistically significant improvement and the LS was superior to the FS.

Magnitude of effect was calculated for pain scores. The standardized mean difference for pain with the FS was 0.43, and 0.79 with the LS. With regards to splint preference the LS was ranked as most preferred by 72% of patients. Of the remaining patients, 16% preferred the FS and 12% indicated no preference for either splint.

## Discussion

The objective for this trial was to determine whether splints relieve pain, and increase perceived function in patients with chronic wrist pain. Of particular interest was the hypothesis that the LS would be more effective than the FS, as noted anecdotally in clinical practice. Findings suggest that both the LS and FS can safely be used in the conservative management of chronic wrist pain in OA and RA, due to lack of adverse effects on stiffness and grip strength, and improvement in patient perceived function. Furthermore, it conclusively demonstrates a significant improvement in patient perceived performance and satisfaction whilst using a splint.

Both splints reduced pain, increased grip strength adding supportive evidence to Haskett's study [6] and the Cochrane review finding that wrist splints are effectual in management of wrist pain [3]. That both splint styles provided a significant improvement in patient perceived function provides supportive evidence to the trends that Pagnotta [8] and Haskett [6] demonstrated.

The results demonstrate a trend for the LS to be more effective than the FS in all AUSCAN measures (pain, stiffness and function) and in grip strength testing. The LS demonstrated the greatest benefit at increasing patient perceived functional performance and satisfaction, measured by the COPM. This functional change was not demonstrated by Haskett [6]. Majority patient preference for the LS supports the significant result for the LS on the COPM, which describes individual subjective experiences. These results add evidence to the findings by Haskett in which a custom-made leather splint provided greatest pain relief compared to commercially available splints, and was also the most preferred style of splint [6]. The authors postulate that the superior effects of the LS are due to its "skin like" fit that provides for both movement and stability during functional performance. Neither the AUSCAN nor COPM questionnaires have previously been used for wrist pain trials but appear sensitive to change based on our results.

The inclusion of a joint stiffness measure allowed for the assessment of a potential adverse effect of splinting. Neither splint increased joint stiffness indicating that use of a wrist splint during patient chosen functional activity does not exacerbate joint stiffness in patients with wrist pain. This may have been due to the fact that there was no minimum wear-time specified, so patients were able to remove the splint and exercise their joints as they saw fit to prevent stiffness.

The calculation of magnitude of effect enables comparison with the benefit of other treatment modalities. Both splint styles produced positive effect sizes on pain. The LS effect size indicates a large clinically significant treatment benefit (0.79), the FS effect size is small to moderate (0.43) [17]. These effect sizes are comparable to exercise therapy on pain in OA (0.58) [18,19], non-steroidal anti-inflammatory drugs on pain in OA and RA (0.66) [18] and greater than the treatment effect of education on pain in OA and RA (0.17) [18]. Importantly, in this trial, the LS provided a greater clinical benefit than all the above-mentioned modalities on pain.

The study is limited in its ability to assess for a carry-over effect due to the lack of assessment of outcomes following the washout period. In addition, there is no supporting literature that provides the optimal length of a wash out period. There is also the potential for a honeymoon effect influencing the patient's subjective responses to the first splint phase. The blinding of the outcome assessor is a notable strength of the study design. The sample size was sufficient for comparisons to baseline but not consistently for comparisons of the LS and FS and subgroup analyses but it is reasonable to conclude that the results can be generalized to a population of adults with chronic wrist pain.

## Conclusion

Therapists must carefully consider the type of splint prescribed, and achieving optimal splint fit and comfort has been shown to be important in achieving pain reduction and functional improvement. This trial supports Haskett's [6] finding that custom-made leather splints are superior to a commercially available fabric splint for short-term relief of wrist pain and dysfunction.

## Competing interests

The authors declare that they have no competing interests.

## Authors' contributions

RN, JT and GJ drafted the manuscript. JR and WR conceived of the study, participated in its' design and coordination. WR was a blinded assessor. GJ and SQ performed the statistical analysis. All authors read and approved the final manuscript.

## Pre-publication history

The pre-publication history for this paper can be accessed here:


